# Evaluation of activities and instrumental activities of daily living and correlated factors of traumatic brain injury patients in Vietnam

**DOI:** 10.1038/s41598-024-58472-2

**Published:** 2024-04-09

**Authors:** Anh Thuy Hoang, Tung Hoang Tran, Hai Minh Vu, Hoa Thi Do, Thuc Minh Thi Vu, Linh Gia Vu, Cuong Tat Nguyen, Huyen Phuc Do, Carl A. Latkin, Roger C. M. Ho, Cyrus S. H. Ho

**Affiliations:** 1 Institute for Preventive Medicine and Public Health, Hanoi Medical University, Hanoi, 100000 Vietnam; 2 Institute of Orthopedic and Trauma Surgery, Vietnam – Germany Hospital, Hanoi, Vietnam; 3 Department of Trauma, Thai Binh University of Medicine and Pharmacy, Thai Binh, 410000 Vietnam; 4 Institute of Health Economics and Technology (iHEAT), Hanoi, 100000 Vietnam; 5 Institute for Global Health Innovations, Duy Tan University, Da Nang, 550000 Vietnam; 6 Faculty of Medicine, Duy Tan University, Da Nang, 550000 Vietnam; 7 Bloomberg School of Public Health, Johns Hopkins University, Baltimore, MD 21205 USA; 8 Department of Psychological Medicine, Yong Loo Lin School of Medicine, National University of Singapore, Singapore, 119228 Singapore; 9 Institute for Health Innovation and Technology (iHealthtech), National University of Singapore, Singapore, 119077 Singapore

**Keywords:** Activities of daily living, Instrumental activities of daily living, Traumatic brain injury, Patients, Vietnam, Trauma, Public health

## Abstract

Traumatic brain injury (TBI) is among the leading causes of death in Vietnam. Survivors of TBI suffer from functional and cognitive deficits. Understanding that Activities of Daily Living (ADLs) and Instrumental Activities of Daily Living (IADLs) are crucial in measuring the treatment and health-related quality of life among patients with TBI. This study aims to evaluate ADLs and IADLs among the TBI population in Vietnam and determine the correlated factors to these two indices. A cross-sectional study was conducted on 212 patients with TBI in Vietnam from February to September 2020. ADLs and IADLs scales were applied. Depression, quality of sleep, and social support scales were used. Multivariate Tobit regression was adopted to identify factors associated with ADLs and IADLs. Patients who received first aid had higher ADLs scores than those who had not, by a statistical difference with a *p* value = 0.04. The mean ADLs score was 5.4 (SD = 1.4). The mean score of IADLs was 7.3 (SD = 1.7). Female patients (Mean = 7.6, SD = 1.1) performed better in IADLs than male patients (Mean = 7.1, SD = 1.9). Both ADLs and IADLs were affected strongly by depression and Injury Severity scores (*p* < 0.01), whereas IADLs were significantly correlated to caregiver types and quality of sleep (*p* < 0.01). Family support was observed as a negatively correlated factor to IADLs. Findings from the study provided evidence for authorities to adjust the health strategies among patients with TBI. Proper prehospital care, a basic low-cost hospital care model, and mental health counseling services should be considered when developing health interventions in Vietnam.

## Introduction

Brain injuries are often considered the most fatal type of injury, affecting approximately 74 million people annually, causing death, disability, and unmeasurable burdens to the victims and society^1^. Traumatic brain injury (TBI) patients suffer from deterioration of health both physically and cognitively, such as partial paralysis, motion impairments, changes in behavioral and emotional functions, or problems with concentration and mind–body cooperation^2,3^. The long-term morbidities caused by TBI lead to loss of working ability, earning capacity, and eventually severe socioeconomic setbacks for the patients and their families^4^.

Activities of daily living (ADLs) include fundamental skills for basic physical needs in everyday life, such as personal care, mobility, and eating^5–7^. Instrumental Activities of Daily Living (IADLs) comprise more complex tasks such as medication, shopping, and financial management^8–10^. Basic and instrumental activities of daily living among individuals with traumatic brain injuries have been studied in several papers. Most studies found that individuals with TBI often experience impairments in executive functions that impact their ability to perform ADLs/IADLs^11–13^. Traumatic Brain Injury causes a range of disabilities that can drastically change a person's life in several ways. TBI can affect daily work and decision-making by causing cognitive problems such as memory impairments, difficulties focusing and paying attention, and interruptions in executive functioning. Physical disabilities, including problems with motor skills and speech or language impairments, can make daily tasks involving physical coordination even more challenging.

Behavioral and emotional changes, such as mood disorders and altered emotional regulation, may impact an individual's general well-being and social interactions. The difficulties associated with vision and hearing impairments might complicate day-to-day tasks. Energy levels and cognitive performance are impacted by fatigue and sleep disruptions, which are prevalent. TBI sufferers may also have headaches, light or sound sensitivity, and, in rare circumstances, seizures. Other studies highlighted the need for research and clinical practice to systematically evaluate basic and instrumental ADL in individuals with post-traumatic stress disorder (PTSD)^14–16^. Individuals who have suffered from traumatic brain damage are more likely to struggle with mental health problems. Patients with traumatic brain injuries, even those with minor wounds, may experience both short- and long-term mental health issues, such as anxiety, depression, and memory loss. TBI is linked to worse health outcomes, such as higher use of mental health services. Research has indicated that compared to those who are not wounded, TBI patients have more excellent rates of heavy drinking and persistent mental health issues^17–19^. Patients with traumatic brain injuries are more likely to seek mental health treatments, indicating a need for care and assistance. Following TBI, mental health issues such as major depressive disorder and post-traumatic stress disorder are frequently noted and may have a role in the persistence of post-concussion symptoms. It is critical to recognize and treat these mental health conditions in TBI patients to improve.

A qualitative study focused on the re-education of specific instrumental activities in patients with severe TBI and found that individualized, contextualized, and intensive rehabilitation can improve their abilities in daily tasks^15^. Additionally, a study investigating the interrelationship of ADLs/IADLs in individuals with moderate to severe TBI found evidence of a hierarchy of skills, with independence in instrumental ADL being associated with independence in basic ADL^12^. Assessments of ADLs and IADLs are used to evaluate the personal well-being of an independent adult through performing basic survival actions and the health outcome of patients after medical treatment or rehabilitation^5^. In global studies, direct assessment of these indicators is often included as one of the best predictors of one's ability to function independently and need for support. As survivors of TBI suffer from severe impairments, their capacity to perform ADLs and IADLs is also severely affected and thus require long-term and intensive care to ensure basic living quality. These kinds of assistance impose serious burdens not only on the patients and their families but also on the healthcare system and the economy. Therefore, understanding the ability to perform daily activities of TBI patients is especially crucial for public health development.

Despite an alarmingly high rate of more than 70% of trauma-related deaths occurring in low-middle-income countries, there is still a lack of data from these countries^20^. In Vietnam, TBI consistently ranks among the top five leading causes of death and accounts for 11% of deaths in 2018^21^. More importantly, among the limited research on TBI impacts on daily life, very few have included the assessment of ADLs as an instrumental measure. Therefore, with the hypothesis: "the degree of TBI is inversely associated with the capacity to conduct both ADLs and IADLs, with persons who have had more severe injuries having larger impairments in everyday functioning," this study was conducted to evaluate the performance of ADLs and IADLs along with other scales among hospitalized TBI patients following their discharge in Vietnam in 2020 and identify factors associated with their performance during recovery.

## Results

Table 1 describes the socioeconomic characteristics of 212 participants in this study. Most respondents were male (67.4%), and the average age of participants was 47.1 years. Respondents living in rural areas accounted for the highest proportion (84.9%). Approximately 70% of respondents were Married/Partnered. The most common occupation was freelancer (42.9%), followed by Blue-collar workers/Farmers (36.8%), and the average monthly income was 469.4 USD (SD = 227.8). In terms of behavior, 23.1% of the study subjects were classified as hazardous alcohol consumers and 76.9% as current smokers. Most of the patients who have traumatic brain injury are had first aid (65.09%).

**Table 1:** Demographic characteristics and health behavior of participants (n = 212).

Characteristics	First aid				Total		*p* value
	No		Yes				
	n	%	n	%	n	%	
Total	74	34.91	138	65.09	212	100.00	
Gender							
Male	52	70.27	91	65.94	143	67.45	0.521
Female	22	29.73	47	34.06	69	32.55	
Current location							
Urban	14	18.92	18	13.04	32	15.09	0.255
Rural	60	81.08	120	86.96	180	84.91	
Education							
Primary/Junior high school	39	52.70	62	44.93	101	47.64	0.472
High school or lower	24	32.43	45	32.61	69	32.55	
Intermediate/college/vocational education	7	9.46	15	10.87	22	10.38	
Tertiary or upper	4	5.41	12	8.70	16	7.55	
Uneducated	0	0.00	4	2.90	4	1.89	
Marital							
Single	16	21.62	33	23.91	49	23.11	0.194
Married/partnered	50	67.57	99	71.74	149	70.28	
Divorced/separated/widowed	8	10.81	6	4.35	14	6.60	
Occupation							
Freelance work	33	44.59	58	42.03	91	42.92	0.928
White-collar workers	7	9.46	12	8.70	19	8.96	
Blue-collar workers/farmers	25	33.78	53	38.41	78	36.79	
Students/unemployment	9	12.16	15	10.87	24	11.32	
Hazardous drinking	15	20.27	34	24.64	49	23.11	0.472
Current smoking	57	77.03	106	76.81	163	76.89	0.972
Comorbidities							
None of the comorbidities	56	75.68	101	73.19	157	74.06	0.892
1 comorbidity	13	17.57	28	20.29	41	19.34	
2 or more comorbidities	5	6.76	9	6.52	14	6.60	
Caregiver							
Parents/spouse	54	72.97	101	73.19	155	73.11	0.973
Offspring	29	39.19	45	32.61	74	34.91	0.338
Grandchildren/helpers	12	16.22	8	5.80	20	9.43	0.013

	Median	p25-p75	Median	p25-p75	Median	p25-p75	*p* value
Age	51	36–61	46	31–60	48	32–60.5	0.194
Monthly income	430.98	301.69–560.27	430.98	301.69–646.47	430.98	301.69–603.37	0.466

Table 2 reports the clinical and social support of participants. The leading causes of TBI were traffic accidents (65.6%) and falls (20.3%). Most participants suffered mild injuries (93.9%) and multiple injuries (62.2%). While two-thirds of patients experienced worsening sleep quality (63.7%), only a tiny proportion of them suffered from moderate or more severe depression (%, ko thấy trong bảng). The average hospitalization time was 11.6 days (SD = 9.3). Support for participants was often provided by the family (5.4 points) compared to that of friends and particular persons (4.7 and 5.0 points, respectively).

**Table 2:** Clinical and social support of participants (n = 212).

Characteristics	First aid				Total		*p* value
	No		Yes				
	n	%	n	%	n	%	
Cause of TBI							
Fall	16	21.62	27	19.57	43	20.28	0.808
Traffic accidents	49	66.22	90	65.22	139	65.57	
Others	9	12.16	21	15.22	30	14.15	
Severity of TBI							
Mild TBI	69	93.24	130	94.20	199	93.87	0.781
Moderate/severe TBI	5	6.76	8	5.80	13	6.13	
Depression							
Minimal or none	56	75.68	110	79.71	166	78.30	0.497
Mild/moderate/moderately severe	18	24.32	28	20.29	46	21.70	
Quality of sleep							
Normal	55	74.32	97	70.29	152	71.70	0.534
Poor sleep	19	25.68	41	29.71	60	28.30	
TBI combined with other injuries	31	43.06	99	72.26	130	62.20	< 0.01
Sequelae	19	29.23	51	40.48	70	36.65	0.126

	Median	p25-p75	Median	p25-p75	Median	p25-p75	*p* value
ISS score	9	6–14	9	3–13	9	6–14	0.304
Social support							
Family group	5	5–6	5	5–6	5	5–6	0.704
Friend group	5	4.25–5	5	4–5	5	4–5	0.614
Special person	5	4.74–6	5	4.5–5.75	5	4.75–6	0.229
Time since being discharged from hospital (months)	4	2–5	3	2–5	3	2–5	0.077
Time in hospital (days)	12	7–15	11	7–14	11	7–14	0.284

Table 3 reports TBI patients' characteristics of Activities and Instrumental Activities of Daily Living. Only 75.9% of patients maintained the full function of Activities of Daily Living. Patients who received first aid before treatment had a better performance of ADLs than those who had not, as indicated by a statistical difference with *p* value = 0.04. The mean ADLs score was 5.4 (SD = 1.4). For the IADL scale, the mean score was 7.3 (SD = 1.7), in which female patients (Mean = 7.6, SD = 1.1) had higher scores than male patients (Mean = 7.1, SD = 1.9).

**Table 3:** Characteristics of activities and instrumental activities of daily living of participants.

Characteristics	First aid				Total		*p* value
	No		Yes				
	n	%	n	%	n	%	
Activities of daily living index							
Full function/moderate impairment	50	67.57	111	80.43	161	75.94	0.037
Severe functional	24	32.43	27	19.57	51	24.06	

	Median	p25-p75	Median	p25-p75	Median	p25-p75	*p* value
ADLs score (range 0–6)	6	5–6	6	6–6	6	6–6	0.027
IADLs score (range 0–8)	8	7–8	8	8–8	8	8–8	0.142

Table 4 suggests that female patients had higher function of ADLs (OR = 5.74, 95% CI = 1.69; 19.53), (Coef. = 1.33; 95% CI = 0.02; 2.64) and IADLs (Coef. = 2.69, 95% CI = 1.31; 4.08) than male patients. Patients who had a caregiver were parents/spouse also had a higher IADLs score (Coef. = 3.83, 95% CI = 2.21; 4.08) and ADL Index (OR = 5.37, 95% CI = 1.74; 16.60) score than those who had not. Higher depression scores and higher ISS scores also corresponded with lower ADLs and IADLs scores. Supporting from friends was a positive factor in IADLs score (Coef. = 1.25; 95% CI = 0.48; 2.02); by contrast, participants who had higher support from family were likely to have a lower IADLs score (Coef. = − 1.65; 95% CI = − 2.57; − 0.73).

**Table 4:** Multivariate Tobit regression for identifying factors associated with activities and instrumental activities of daily living of participants.

Factors	IADLs score		ADLs index		ADLs score	
	Coef	95%CI	OR	95%CI	Coef	95%CI
Socio-economic						
Age (unit: year)	− 0.05**	− 0.09; − 0.00	0.95***	0.92; 0.98	− 0.08***	− 0.12; − 0.04
Gender (female vs male—ref)	2.69***	1.31; 4.08	5.74***	1.69; 19.53	1.33**	0.02; 2.64
Caregiver (yes vs no—Ref)						
Parents/spouse	3.83***	2.21; 5.46	5.37***	1.74; 16.60	1.22*	− 0.06; 2.50
Offsprings	1.45*	− 0.14; 3.03				
Current location (Rural vs urban areas -Ref)	0.95	− 0.49; 2.39				
Occupation (vs freelance worker—ref)						
White-collar workers	0.48	− 2.07; 3.03				
Blue-collar workers/farmers	0.60	− 0.63; 1.82				
Students/unemployment	− 1.96**	− 3.74; − 0.19				
Hazardous drinking (yes vs no—ref)	1.42*	− 0.26; 3.11	2.30	0.67; 7.91		
Current smoking (yes vs no—ref)	− 1.30	− 2.89; 0.29				
Clinical characteristics						
First aid (yes vs no—ref)					0.88	− 0.24; 2.00
Comorbidities (vs. None of the comorbidity—ref)						
1 comorbidity	− 1.10	− 2.46; 0.25	0.60	0.19; 1.84		
2 or more comorbidities	0.33	− 1.56; 2.22	4.20	0.73; 24.31		
Cause of TBI (vs Fall—Ref)						
Traffic accidents	− 0.33	− 1.73; 1.08				
Others	2.35*	− 0.17; 4.88				
TBI combined with other injuries (yes vs no ref)	1.75***	0.51; 2.99				
Severity of TBI (Mild TBI vs moderate/severe TBI—Ref)	1.22	− 1.19; 3.63				
ISS score (unit: score)	− 0.13***	− 0.22; − 0.04	0.87***	0.80; 0.94	− 0.15***	− 0.23; − 0.07
Depression (mild/moderate/moderately severe vs minimal or none—ref)	− 3.88***	− 5.35; − 2.41	0.04***	0.01; 0.15	− 3.56***	− 4.88; − 2.24
Interaction						
Measurement of perceived social support						
Family group (unit: score)	− 1.65***	− 2.57; − 0.73				
Friend group (unit: score)	1.25***	0.48; 2.02				

***p < 0.01; **p < 0.05; *p < 0.1

## Discussion

This study offers insight into the evaluation of ADLs and IADLs among patients with TBI. A high score of ADLs and IADLs among people suffering from traumatic brain injuries was reported in our results. Patients who have caregivers, suffer from depression, or have combined injuries, having social support were associated with the activities of daily living and instrumental activities of daily living of patients.

In this study, a higher percentage (65%) of participants have received first aid compared to prior studies on first aid status in Vietnam^22^. An association between prehospital care and health outcomes after treatment has been indicated in many previous studies^23,24^, and is also consistent with our results, as shown by a statistical difference *p* value of 0.04. While much evidence has been given on the importance of first aid for a patient's chance of survival and long-term health outcomes, little effort has been directed toward improving the capacity of prehospital care in Vietnam. From a public health viewpoint, it is urgent that basic first-aid training for trauma care be provided at a young age. Although first-aid training has long been integrated into all levels of education, the scale and severity of injuries taught were insufficient for real-life emergencies. As shown by our study, the leading cause of TBI in Vietnam is traffic accidents (65.6%). However, instructions on how to relieve blood pressure or to minimize bone fractions are rarely, if not never, included in first aid lessons. Most first-aid training at schools and institutions was also provided theoretically—as lessons, and occasionally—2 to 3 lessons per school year/working year, meaning people cannot gain the experience nor exposure adequate for practical application. As a result, traffic accident patients are often transported to hospitals without proper first aid, which significantly affects survival rate and treatment outcomes^22^.

Interestingly, family support was shown to be negatively correlated with IADLs. Professional caregivers might offer specialized aid, but family caregivers may offer practical and emotional assistance. Training, availability, and skills fitting with the needs of the tasks at hand are factors that affect how well the caring arrangement supports IADLs. Improving the assistance given to IADLs and raising the general quality of life for individuals receiving care requires a cooperative and well-coordinated strategy that includes open communication between caregivers, care recipients, and other stakeholders. There has been inconsistency about the influence of family care on patients with low IADLs performance. A previous study indicated a positive relationship between family support and the independence of care recipients^25^, while others suggested that family assistance reduced the patients' independence and forbade long-term IADLs recovery^26^. Indeed, there is no official model and correlation between family support and TBI recovery, as this factor is entirely based on the dynamic of each patient's family. While family members are not always the most suitable caregivers, they are often the most convenient and, in many cases, the only choice of care provider for most patients. As indicated, most TBI patients were freelancers or farmers, occupations with only low to average income. Low budget combined with high out-of-pocket costs of treatment and surgery means that most TBI patients cannot hire professional caregivers and have to resort to family members, which in turn amplifies the physical and mental burden for both the patients and their families. To alleviate this problem, Vietnamese hospitals should consider adopting hospital care services similar to those in developed countries. In recent years, primary low-cost hospital and home care services by retired nurses^27^ have been increasingly popular as they provide immediate patient benefits and create employment opportunities for the community. This model is especially suitable for patients with low ADLs and IADLs performance, as a health caregiver can easily carry out essential daily living tasks to cater to the needs of such patients. Therefore, Vietnamese hospital authorities should consider adopting a basic low-cost hospital care model, not only to alleviate possible patient-family tension but also to avoid nonclinical factors affecting recovery and improve long-term treatment outcomes.

Our study has shown that the execution of instrument activity of daily living among patients is severely impacted by the impact of numerous traumas, including damage to different body parts in addition to a traumatic brain injury. Complicating the performance of IADLs is the mix of physical injuries that cause pain, discomfort, and motor deficits. Planning and doing everyday tasks are further hampered by cognitive issues resulting from traumatic brain injury, such as memory and concentration difficulties. A thorough rehabilitation strategy considering cognitive and physical components is required since functional independence is frequently hampered. When it comes to specialized interventions in physical therapy, occupational therapy, and mental health assistance, the rehabilitation process becomes noticeably complex and requires cooperation among healthcare specialists. The psychosocial effects extend beyond the physical domain and affect mental and emotional well-being. The complexities of care put more demands on caregivers, and it becomes necessary to make home adaptations, adaptive tactics, and assistive technology to foster independence. In general, enhancing functional results and assisting persons in their journey towards regaining independence in everyday activities require tackling the complex problems brought about by various traumas.

Finally, mental counseling is recommended for psychological well-being and social integration. Immobility and the inability to change the environment are everyday stressors and can lead to severe mental health issues. In Vietnamese hospitals, although psychological problems among trauma patients are well-evident, not much consideration has been given to mental therapy for clinical patients. The lack of professional, intensive mental support is hazardous in the hospital's depression-prone setting. As our results suggested, a large proportion of participants suffered from worsening mild depression. Therefore, more efforts should be invested into mental healthcare, from professional services to simple stress-alleviating activities such as playing radio music or group talk and counseling.

The results of this study provide the first evaluation of ADLs and IADLs among patients with TBI in Vietnam and highlight factors associated with ADLs and IADLS performance. The entire function of ADLs and high scores in ADLs and IADLs indicated improvement in quality in performing vital self-care tasks and independence to live in the community after hospitalization discharge. Trends in the performance of such tasks provide insights into the status of TBI patients and evidence for the development of health interventions in Vietnam. This study had several limitations. First, according to the study design, all variables are evaluated after the treatment. Therefore, there is no available comparison of life quality before and after the intervention. Secondly, self-report accuracy is not guaranteed. Recall that bias and social perception may cause specific answers to be underestimated or overestimated. Finally, convenience sampling techniques may have restricted the applicability of the findings to the Vietnamese population.

## Conclusion

As a result of rapid industrialization and urbanization, Vietnam is experiencing a rising number of traumatic brain injury cases, creating a tremendous financial, physical, and mental burden to the patients, families, and the country. Evaluation of ADLs and IADLs among the TBI population offers evidence to implement timely interventions to reduce any nonclinical problems during recovery and enhance patients' long-term independence. Our findings provide policymakers insights into adjusting health policies and healthcare services for people with TBI. Similarly, these findings can be utilized as benchmarks by health departments to assess the disparity in quality of life between TBI patients and other population groups in Vietnam.

## Methods

### Study design and participants

A cross-sectional study was conducted on 212 patients at the Department of Neurosurgery Spine of Thai Binh Provincial General Hospital (Vietnam) from February to September 2020. Patients who were recruited in this study had the following criteria: (1) had undergone traumatic brain injury treatment in the Department of Neurosurgery Spine of Thai Binh Provincial General Hospital, (2) were aged above 18, and (3) were currently living in Vietnam. Individuals with significant cognitive impairments or those incapable of responding to data collectors' inquiries were not considered for recruitment.

### Measure and instruments

A 20-min telephone interview was conducted on each patient. The telephone interview was adopted instead of other approaches because it is low-cost, less time-consuming, and easy to follow. The patient was called for an interview 1 to 8 months after discharge from the hospital. Investigators underwent thorough training to administer phone-based questionnaire interviews. The validity of the survey questions was confirmed by conducting a pilot survey among 10 participants with varying ages, genders, and occupations. The semi-structured questionnaire was created and consisted of the following primary sections:

#### Socioeconomics

Respondents answered basic demographic questions: Gender, Occupation, Educational level, Marital status, Average monthly household income ($US), and Caregiver status.

#### Health risk behavior

To evaluate the health risk behavior, we asked respondents about their usage of cigarettes and alcohol. Specifically, we surveyed the proportion of respondents who smoked in the last 30 days. Survey questions about alcohol consumption were developed from the global Alcohol Use Disorders Identification Test (AUDIT-C). They included three questions with an overall score of 12 points: frequency of general alcohol consumption frequency, amount of alcohol consumption per day, and frequency of heavy (6 or more drinks) alcohol consumption^28^. An AUDIT-C score below 4 points (male) and 3 points (female) were considered positive.

#### Clinical characteristics

To present the characteristics of Traumatic Brain Injury (TBI), we asked participants about the cause and severity of their injury and types of other simultaneous injuries. Furthermore, four measurement scales were adopted to assess patients' clinical characteristics, physical and mental.

- *Injury Severity Score (ISS)* We used ISS to evaluate trauma severity based on injury of 6 body systems (Head/Neck, Face, Chest, Abdomen, Extremities, and External). Specific injuries in each body region are coded on a scale of 1 (minor), 2 (moderate), 3 (serious, not life-threatening), 4 (severe, life-threatening, survival probable), 5 (critical, survival uncertain) and 6 (unsurvivable). The data was collected through participants' medical records after obtaining the patients' and doctors' agreement. The score ranged from 0 to 75^29^. If an injury is assigned an AIS of 6 (identifying a currently untreatable injury), the ISS score is automatically assigned 75^29^.
- *Patient Health Questionnaire-9 (PHQ-9)* We used PHQ-9 to evaluate the mental health status of respondents. This scale had nine items; each item was scored from 0 to 3, and the total score ranged from 0 to 27^30^. 5 levels of mental status are Minimal depression (1–4), Mild depression (5–9), Moderate depression (10–14), Moderately severe depression (15–19), Severe depression (20–27)^30^. The Cronbach's alpha was good at 0.8218.
- *Pittsburgh Sleep Quality Index (PQSI)* We used PQSI to evaluate respondents' sleep quality. Seven domains were assessed, including subjective sleep quality, latency, duration, habitual sleep efficiency, sleep disturbances, use of sleeping medication, and daytime dysfunction. Each item was scored from 0 to 3, and the total score ranged from 0 to 21^31^. Two levels of the PSQI scale are poor sleep (total score ≥ 5; a higher score indicated the worse quality of sleep) and normal sleep (total score of < 5)^31^.
- *The Measurement of Perceived Social Support Scale (MSPSS scale)* We used the MSPSS scale to evaluate the social support received by respondents. This section was divided into three groups: Family, Friends, and Significant Others^32^. Each group was scored from 1 to 7, and a higher score indicated more support was provided for patients^32^. Higher scores indicated higher support from family, friends, or significant others^32^. The maximum total score was 84, with a higher score indicating better perceived social support. The Cronbach's alpha was 0.9592.

To evaluate the traumatic brain injury patients' ability to complete activities of daily living, which is the outcome of research, we used two scales:

- *Katz Index of Independence in Activities of Daily Living (ADL)* We used the ADL scale to assess the ability to perform activities of daily living independently of respondents^6,7^. This tool assesses the Basic ADL function among older adults in the community and all care settings. Six items included bathing, dressing, transferring, toileting, feeding, and continence. To score this tool, if a respondence can perform an activity, he/she gets a score of 1, and if he/she cannot do so, will get a score of 0. The total score varies between 6 (maximum performance) and 0 (lack of performance). A score of 6 indicates full function, 4 indicates moderate impairment, and two or less indicates severe functional impairment^6,7^. The Cronbach's alpha of the ADL scale in this study was 0.9272.
- *Lawton Instrumental Activities of Daily Living Scale (IADL)* We used the IADL scale to assess more complex daily activities and independent living skills^9,10^. Eight items included the ability to use the telephone, shopping, food preparation, housekeeping, laundry, mode of transportation, responsibility for own medications, and ability to handle finances. The scoring scale is zero and one, and the sum of the scores varies from 0 (low function, dependence) to 8 (high function, independence)^9,10^. The Cronbach's alpha of the IADL scale in this study was 0.9357.

### Statistical analysis

Data were analyzed using STATA version 16 (Stata Corp. LP, College Station, United States of America). With missing data, we used the Listwise Deletion method to clean data before analyzing. We used the Chi-squared and Kruskal–Wallis Tests to analyze participant demographic characteristics, health risk behaviors, and clinical characteristics. Multivariate Tobit regression was used to determine factors related to the IADLs and ADLs scores. ADLS index-associated factors were analyzed in the multivariate logistic regression model. A forward stepwise selection method was employed to eliminate unimportant factors, with a threshold of 0.2. Any *p* value less than 0.05 was deemed statistically significant.

### Ethical considerations

The study protocol was approved by the Institutional Review Board of Thai Binh University of Medicine and Pharmacy (Code: 511/QD-YDTB). Informed written consent was obtained from all participants. Participants could refuse to participate at any time without any impact on their treatment. Their data was kept safe, and only the principal investigators could access it. All methods were performed following the relevant guidelines and regulations.

## Data Availability

The datasets used and/or analyzed during the current study are available from the corresponding author upon reasonable request.

## References

[1] GBD 2016 Traumatic Brain Injury Spinal Cord Injury Collaborators (2019). Global, regional, and national burden of traumatic brain injury and spinal cord injury, 1990–2016: A systematic analysis for the Global Burden of Disease Study 2016. Lancet Neurol..

[2] Azouvi P, Arnould A, Dromer E, Vallat-Azouvi C (2017). Neuropsychology of traumatic brain injury: An expert overview. Rev. Neurol. (Paris).

[3] Cheng P (2017). Trends in traumatic brain injury mortality in China, 2006–2013: A population-based longitudinal study. PLoS Med..

[4] Nguyen R (2016). The international incidence of traumatic brain injury: A systematic review and meta-analysis. Can. J. Neurol. Sci..

[5] Edemekong, P. F., Bomgaars, D. L., Sukumaran, S. & Levy, S. B. Activities of daily living. In *StatPearls* (2022). 29261878

[6] Katz S, Ford AB, Moskowitz RW, Jackson BA, Jaffe MW (1963). Studies of illness in the aged. The index of Adl: A standardized measure of biological and psychosocial function. JAMA.

[7] Katz S, Downs TD, Cash HR, Grotz RC (1970). Progress in development of the index of ADL. Gerontologist.

[8] Guo, H. J. & Sapra, A. Instrumental activity of daily living. In *StatPearls* (2022). 31985920

[9] Lawton MP, Brody EM (1969). Assessment of older people: Self-maintaining and instrumental activities of daily living. Gerontologist.

[10] Edemekong, P. F., Bomgaars, D. L., Sukumaran, S. & Schoo, C. *Activities of Daily Living* (StatPearls Publishing, Treasure Island (FL), 2022). 29261878

[11] Ertas-Spantgar F, Korabova S, Gabel A, Schiering I, Muller SV (2022). Guiding patients with traumatic brain injury through the instrumental activities of daily living with the RehaGoal App: A feasibility study. Disabil. Rehabil. Assist. Technol..

[12] Giles GM, Clark-Wilson J, Baxter DM, Tasker R, Holloway M, Seymour S (2019). The interrelationship of functional skills in individuals living in the community, following moderate to severe traumatic brain injury. Brain Inj..

[13] Wilson L (2020). Understanding the relationship between cognitive performance and function in daily life after traumatic brain injury. J. Neurol. Neurosurg. Psychiatry.

[14] Punski-Hoogervorst JL, Avital A, Engel-Yeger B (2023). Challenges in basic and instrumental activities of daily living among adults with post-traumatic stress disorder: A scoping review. Occup. Ther. Ment. Health.

[15] Kanchan A, Singh AR, Khan NA, Jahan M, Raman R, Sathyanarayana Rao TS (2018). Impact of neuropsychological rehabilitation on activities of daily living and community reintegration of patients with traumatic brain injury. Indian J. Psychiatry.

[16] Khan F (2016). Factors associated with long-term functional and psychological outcomes in persons with moderate to severe traumatic brain injury. J. Rehabil. Med..

[17] Fahmy C, Testa A, Jackson DB (2023). Traumatic brain injury and mental health outcomes among recently incarcerated men. J. Trauma. Stress.

[18] Kureshi N, Clarke DB, Feng C (2023). Association between traumatic brain injury and mental health care utilization: Evidence from the Canadian Community Health Survey. Inj. Epidemiol..

[19] Shi S, Almklov E, Afari N, Pittman JOE (2023). Symptoms of major depressive disorder and post-traumatic stress disorder in veterans with mild traumatic brain injury: A network analysis. PLoS ONE.

[20] Tropeano MP (2019). A comparison of publication to TBI burden ratio of low- and middle-income countries versus high-income countries: How can we improve worldwide care of TBI?. Neurosurg. Focus.

[21] World Health Organization (WHO). World health statistics 2017: Monitoring health for the SDGs (2017).

[22] Mai HT (2020). The status of first aid and its associations with health outcomes among patients with traffic accidents in urban areas of Vietnam. Int. J. Environ. Res. Public Health.

[23] Myburgh JA (2008). Epidemiology and 12-month outcomes from traumatic brain injury in australia and new zealand. J. Trauma.

[24] Wu X (2008). Epidemiology of traumatic brain injury in eastern China, 2004: A prospective large case study. J. Trauma.

[25] Sigalingging G, Sitopu SD (2017). Measuring family support in the Elderl™ s independence in performing activities of daily living (a case study in Medan Tuntungan District, Medan City, Indonesia). IOSR J. Nurs. Health Sci..

[26] Fang Y (2017). Patient and family member factors influencing outcomes of poststroke inpatient rehabilitation. Arch. Phys. Med. Rehabil..

[27] UNC Health Talk. Retired Nurse Benefits from Hospital-at-Home Program (2021).

[28] Bradley KA, DeBenedetti AF, Volk RJ, Williams EC, Frank D, Kivlahan DR (2007). AUDIT-C as a brief screen for alcohol misuse in primary care. Alcohol. Clin. Exp. Res..

[29] Baker SP, O'Neill B, Haddon W, Long WB (1974). The injury severity score: A method for describing patients with multiple injuries and evaluating emergency care. J. Trauma.

[30] Review T (2014). Patient health questionnaire–9 (PHQ-9). Rehabil. Couns. Bull..

[31] Buysse DJ (2008). Relationships between the Pittsburgh Sleep Quality Index (PSQI), Epworth Sleepiness Scale (ESS), and clinical/polysomnographic measures in a community sample. J. Clin. Sleep Med..

[32] Zimet G. *Multidimensional Scale of Perceived Social Support (MSPSS) - Scale Items and Scoring Information* (2016).

